# Associations between habitual walking speed and in vivo tibial cartilage strain in individuals with anterior cruciate ligament reconstruction

**DOI:** 10.1016/j.ostima.2025.100390

**Published:** 2025-12-17

**Authors:** Caroline Lisee, David Lalush, Cortney Armitano-Lago, Elizabeth Bjornsen, Jason R. Franz, Jeffrey T. Spang, Matthew E. Dausch, Brian Pietrosimone

**Affiliations:** a Department of Kinesiology, University of Georgia, 330 River Road, Athens, GA 30605, USA; b Lampe Joint Department of Biomedical Engineering, University of North Carolina at Chapel Hill and North Carolina State University, 10010 Mary Ellen Jones, Campus Box 7575, Chapel Hill, NC 27599, USA; c School of Medicine, University of North Carolina at Chapel Hill, Bondurant Hall, Campus Box 7120, Chapel Hill, NC 27599, USA; d Department of Orthopaedics, University of North Carolina at Chapel Hill, 130 Mason Farm Road, Campus Box 7055, Chapel Hill, NC 27599, USA; e Department of Exercise and Sports Science, University of North Carolina at Chapel Hill, 210 South Road, Campus Box 8700, Chapel Hill, NC, 27599 USA

**Keywords:** Cartilage, articular, Walking speed, Magnetic resonance imaging, Anterior cruciate ligament reconstruction, Osteoarthritis, Knee, Knee Injuries

## Abstract

**Objectives:**

The purpose of the study was to determine associations between habitual walking speed and an in vivo magnetic resonance imagining (MRI) measure of tibial cartilage strain following primary anterior cruciate ligament reconstruction (ACLR).

**Methods:**

Habitual walking speed was collected over ground in 12 individuals (5 Males/7 Females). Participants underwent an MRI of their ACLR knee to capture resting tibial articular cartilage thickness. Following the MRI, participants walked on a treadmill at their habitual walking speed for 3000 steps. Cartilage thickness was calculated for pre-loading and post-loading across the entire articulating surface and strain maps were created by calculating the change in thickness from pre- to post-loading thickness. Average cartilage strain was calculated in 6 evenly divided regions of interest (ROI) of the medial and lateral tibial cartilage. Spearman’s rank correlations and 95 % confidence intervals (CIs) (ρ) were used to assess associations between habitual walking speed and average tibial articular cartilage strain in each ROI.

**Results:**

Slower habitual walking speed was moderately associated with greater strain in medial central inner tibial cartilage region (ρ=-0.67, *95 %CI=[-90,-0.14]*). All other associations between cartilage strain and medial (ρ range= -0.30 to 0.06, *95 %CI* range=-0.75 to 0.62) or lateral (ρ range= -0.42 to 0.28, *95 %CI* range=-0.81 to 0.74) tibial cartilage ROIs were weak to moderate.

**Conclusion:**

Our study demonstrates a measurable in vivo link between slower habitual walking speeds and greater cartilage strain, which provides further rationale for evaluating the mechanistic link between modifying habitual walking speed to influence important biophysical changes in cartilage health.

## Introduction

Osteoarthritis is a disease with no known cure and a leading cause of disability in the United States [1,2]. Traumatic knee injury is a primary risk factor for knee osteoarthritis (KOA) development, as one-third of individuals exhibit radiographic KOA within a decade of anterior cruciate ligament (ACL) reconstruction (ACLR) [3]. Identifying mechanisms associated with KOA development is imperative for developing novel preventive strategies to combat KOA disease onset in people with an ACLR. Habitual walking speed is an important indicator of functional limitations in patients with KOA [4] and is an easily acquired clinical measure that predicts knee confidence [5] and likelihood to undergo a total knee arthroplasty [6]. Slower habitual walking speed is an early biomarker of incidence of KOA in middle-aged and older adults [7,8]. Individuals with KOA who walk at slower habitual walking speeds are more likely than faster walkers to exhibit progressive KOA [8]. More recent work indicates that slower habitual walking speed is associated with deleterious joint tissue changes that are consistent with KOA development in younger patients with an ACLR [9,10], suggesting that behavioral adaptations of slower habitual walking speed post-ACLR may contribute to underlying pathophysiological changes of joint tissue breakdown. Understanding the link between habitual walking speed and progression of joint tissue breakdown post-ACLR may lead to easily scalable screening platforms to identify heightened KOA risk and novel interventions to optimize walking speed for the purpose of lessening the risk of KOA development.

ACLR individuals exhibit slower walking speeds compared to matched (i.e., age, sex, BMI) uninjured controls at 12 months post-ACLR [10]. Individuals who walk slower at 6 and 12 months post-ACLR exhibit worse tibiofemoral articular cartilage proteoglycan density compared to faster walkers at 12 months post-ACLR (i.e., greater T1ρ MRI relaxation times) [10]. Furthermore, those who demonstrate habitual walking speeds ≤1.27 m/s exhibit 3 times higher odds of experiencing clinically significant knee-related symptoms compared to those who walk faster than 1.28 m/s at 6 months post-ACLR [11]. At later time points post-ACLR (43.2 ± 36.4 months post-ACLR), slower habitual walking speed is associated with greater concentrations of serum biomarkers of Type-II collagen breakdown [9]. Overall, these studies demonstrate an observational link between slower walking speeds post-ACLR and early KOA related joint tissue and metabolic changes. However, it remains unclear if slower habitual walking speed is a mechanism contributing to joint tissue breakdown or an indicator of underlying KOA disease development.

It can be hypothesized that slower walking speeds may contribute to harmful articular cartilage mechanics as articular cartilage is viscoelastic and undergoes strain in a time dependent manner [12]. Slower walking speeds are associated with longer stance durations [13] which may exert compressive force to the articular cartilage over a longer time during a single step. Further, slower walking speeds following ACLR are associated with less dynamic compressive limb-level loading that are characterized by vertical ground reaction forces with lower first and second peaks and greater compressive force at midstance [14]. Less dynamic, or more sustained, limb loading is associated with worse cartilage composition and may negatively impact tissue fluid dynamics leading to more cartilage strain during walking [15]. Previous work evaluating a single cross-sectional region of knee articular cartilage using ultrasound reported that slower uninjured controls demonstrated greater cartilage deformation following 30 min of walking [16]. Despite persistent differences in habitual walking biomechanics including speed [10] and worse cartilage composition compared to uninjured controls [17,18], the effect that slower habitual walking speed has on knee cartilage strain in individuals with ACLR remains unknown. Determining the association between habitual walking speed and cartilage strain is important for understanding the potential influence that slower walking speed would have on biophysical properties linked to cartilage health [19]. Such information would have important implications on gait retraining and rehabilitation following ACLR.

Therefore, the purpose of this study was to determine the association between habitual walking speed and tibial articular cartilage strain following a standardized walking protocol in individuals with ACLR. Our study focused on cartilage strain of the tibia because previous research reports greater tibial cartilage strain, but not medial or lateral femoral condylar strain, in the limb of individuals with an ACL injury compared to their uninjured limb [20]. We hypothesized that individuals with slower habitual walking speed would exhibit greater tibial articular cartilage strain after walking.

## Methods

This observational, cross-sectional laboratory study was performed over two sessions. During the first session, we collected demographics, knee injury history information and determined habitual walking speed for each participant. Participants also walked on a treadmill at their habitual walking speed for at least 5 min until they felt comfortable walking on the treadmill for acclimatization to the protocol. During the second session, participants underwent MRI scans of tibial cartilage thickness in the ACLR limb before (i.e., preloading) and after (post-loading) performing a walking protocol at habitual walking speed. Sessions 1 and 2 were completed on separate days at least 24 h apart so the habitual walking speed testing and acclimatization to the protocol did not affect the results of the MRI. Participants were instructed to avoid any strenuous physical activity or any new activities (i.e., out of the ordinary) at least 24 h prior to the second session. Study recruitment and methodology were approved by the university’s Institutional Review Board (IRB# 21–2181), and all participants provided written informed consent before study participation.

### Participants

Participants were recruited as a convenience sample from a local orthopedic clinic and the University’s campus via flyers or emails. Individuals between the ages of 18 to 35 who underwent primary ACLR surgery within 6 to 60 months of enrollment and reported no restrictions to walking were included in the study. Individuals were excluded if they had undergone multiple ACL injuries or surgeries, underwent multi-ligament surgery at the time of ACLR, sustained a lower extremity fracture at the time of the ACL injury, or were diagnosed with any form of arthritis. Participants were not excluded from the study if they had a history of meniscal injury at the time of ACL injury or meniscal-related procedure at the time of ACLR. Table 1 describes the frequency of participants with concomitant meniscal injury or a meniscal-related procedure.

**Table 1:** Participant characteristics (N = 12).

Outcomes	Mean ± Standard Deviation
Age (years)	22.9 ± 3.9
Sex ( % Male/ %Female)	42 %/58 %
Body mass index (kg/m^2^)	25.6 ± 2.7
Months Post-ACLR	29.1 ± 18.0
Graft Type	PT: 67 % (N = 8), HT: 25 % (N = 3), QT: 8 % (N = 1)
Meniscal Injury	58 % (N = 7)
Meniscectomy	25 % (N = 3)
Meniscal Repair	42 % (N = 5)
Habitual Walking Speed (m/s)	1.2 ± 0.2
Treadmill Walking Time (min.)	28.48±1.35
Time between completion of treadmill walking and post-loading MRI (min.)	8.15±2.39

Abbreviations: PT = Patellar Tendon Graft, HT = Hamstring Tendon Graft, QT = Quadriceps Tendon Graft.

### Habitual walking speed

Infrared timing gates (Dashr Motion Performance Systems, Omaha, Nebraska) were placed 0.97 m apart in the middle of a flat 6-meter walkway [10,21]. Participants were instructed to walk across the 6 m walkway between the timing gates as if they were, “comfortably walking on a sidewalk with a purpose” [9,10,14,[21], [22], [23]]. Participants performed practice trials until they reported feeling comfortable with the task. After completing the practice trials, participants completed five trials for assessment which were averaged together to calculate habitual walking speed. The calculated average habitual walking speed was used as the participant’s walking speed for the standardized treadmill walking protocol and as the exposure variable for the statistical analysis.

### Magnetic resonance imaging data collection and processing

Participants sat with their knees unloaded in an extended position for 1 hour before completing the first MRI scan to minimize the effects of loading that occurred earlier in the day [20,24]. Next, participants were transferred to the MRI scanner with an MRI safe wheelchair to minimize steps and eliminate potential weightbearing of the knee. Upon completion of the pre-loading MRI scan, participants were transferred to a treadmill in a wheelchair to complete the standardized treadmill walking protocol. To complete the walking protocol, participants walked on the treadmill for 3000 steps at their habitual walking speed [25,26]. A total of 3000 steps was chosen for the walking protocol as previous studies assessing knee cartilage thickness through ultrasonography found the greatest knee cartilage deformation occurs at 3000 daily steps compared to other step counts (i.e., 1000, 2000, 4000, 5000 steps) [27]. Immediately following the 3000 step protocol, participants were transferred to the MRI scanner in a wheelchair to perform the post-loading MRI scan.

MRI scans were collected using a Siemens PRISM 3T scanner (Siemens, Munich, Germany) with a 15-channel Siemens knee coil (TX/RX Knee Coil 15 Flair 3T). A 3D localizer was used prior to a sagittal 3D double-echo steady state (DESS) sequence which acquired three-dimensional volumes of ACLR limb tibiofemoral joint (field of view = 160 × 160 × 96 mm, slice thickness = 1 mm, voxel size =0.3 × 0.3 × 1 mm, TR/TE = 17/6 ms, FA = 25 deg, phase encoding direction: anterior to posterior, approximate scan time = 8.5 min). Preloading and post-loading images of the tibiofemoral joint were automatically registered with a trained convolutional neural network (CNN). Specifically, the 55-layer U-net CNN, trained on 43 manually-labeled DESS images, labeled the tibia and femur bones. Then, a rigid transformation aligned the post-loading tibia bone to the preloading tibia bone; rigid registration was required so as not to distort cartilage geometry. A single assessor who has demonstrated excellent test-retest (ICC range=0.89–0.99) and intra-rater reliability (ICC=0.84–0.99) manually segmented the tibial articular cartilage on all slices of the pre- and registered post-loading images. The single assessor was blinded to the participant characteristics and habitual walking speed. Articular cartilage segmentations were used to define the geometry of the tibial bone as well as the medial and lateral articular cartilage. The cartilage segmentations were converted to polygonal surface models using Slicer(www.slicer.org).

For both the pre-loading and post-loading images, cartilage thickness was calculated as the distance between closest vertices on the synovial-cartilage surface and the cartilage-bone surface. The cartilage thickness of each vertex was flattened and resampled to 1 mm 2-dimensional (2D) maps in the transverse plane. Cartilage strain ( %) was calculated at each map pixel from the 2D cartilage thickness maps using the following equation to create cartilage strain maps:

$$=\frac{preloading\;cartilage\;thickness-postloading\;cartilage\;thickness}{preloading\;cartilage\;thickness}x\;100$$

**Fig. 1:**
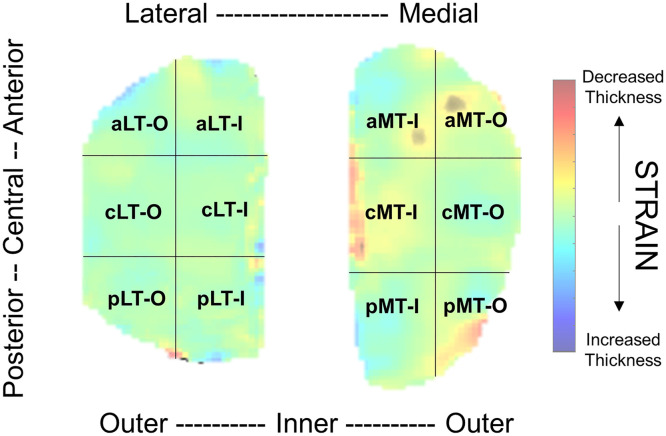
Representative 2D tibial cartilage strain map from a single participant with 12 evenly divided ROIs. Abbreviations: MT = Medial Tibial, LT = Lateral Tibia, O = Outer, I = Inner, a=anterior, c=central, p=posterior.

The 2-mm borders of the medial and lateral cartilage strain maps were removed. The cartilage strain map was evenly divided in half based on the farthest coordinates in the medial/lateral direction and into thirds based on the farthest coordinates in the anterior/posterior direction for both the medial and lateral cartilage, creating 12 total regions of interest (ROIs) (Fig. 1). Mean cartilage strain ( %) was calculated by averaging the cartilage strain across each map pixel in a specified ROI. Larger, positive cartilage strain values indicate larger decreases in tibial cartilage thickness from pre- to post-loading.

### Statistical analysis

Means and standard deviations were calculated for participant characteristics, habitual walking speed and tibial cartilage strain outcomes. Frequencies and percentages were calculated for participant surgical characteristics. Due to the modest sample size [28], Spearman’s ρ correlations and 95 % confidence intervals (CI) were used to determine the associations between habitual walking speed and cartilage strain outcomes. Correlation coefficients were classified as weak (0 – 0.4), moderate (0.41 – 0.7), and strong (≥0.71) [29]. All analyses were performed in SPSS (version 29, IBM Corporation, Armonk NY).

## Results

**Fig. 2:**
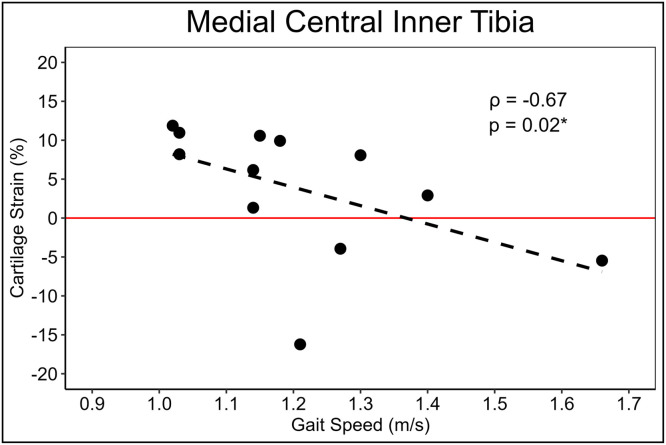
Scatter plot depicting the statistically significant association between habitual walking speed (m/s) and medial central inner tibial cartilage strain ( %).

Participant characteristics and tibial cartilage strain outcomes are reported in Table 1, Table 2, respectively. Slower habitual walking speed was associated with greater cartilage strain in the medial central inner tibial cartilage region and confidence intervals did not cross zero (Spearman’s ρ = −0.67, 95 % CI [−0.90, −0.14] Fig. 2). Habitual walking speed demonstrated weak to moderate associations with tibial articular cartilage strain in all other medial (Spearman’s ρ range = −0.30 to 0.06, 95 % CI range = −0.75 to 0.62) and lateral (Spearman’s ρ range = −0.42 to 0.28, 95 % CI range = −0.81 to 0.74) regions, but the 95 % confidence intervals crossed zero (Table 3).

**Table 2:** Means and standard deviations of Tibial cartilage % strain.

Region of Interest	Mean ± Standard Deviation
Medial anterior outer tibial cartilage (aMT-O)	2.1 ± 7.7
Medial central outer tibial cartilage (cMT-O)	1.7 ± 5.5
Medial posterior outer tibial cartilage (pMT-O)	1.5 ± 7.0
Medial anterior inner tibial cartilage (aMT-I)	4.6 ± 6.1
Medial central inner tibial cartilage (cMT-I)	3.7 ± 8.5
Medial posterior inner tibial cartilage (pMT-I)	2.3 ± 8.8
Lateral anterior outer tibial cartilage (aLT-O)	1.1 ± 7.4
Lateral central outer tibial cartilage (cLT-O)	5.1 ± 7.0
Lateral posterior outer tibial cartilage (pLT-O)	3.7 ± 5.7
Lateral anterior inner tibial cartilage (aLT-I)	2.8 ± 6.9
Lateral central outer inner cartilage (cLT-I)	3.8 ± 4.0
Lateral posterior outer inner cartilage (pLT-I)	2.3 ± 8.8

**Table 3:** Associations between Tibial cartilage strain and habitual walking speed.

Region of Interest	Spearman’s ρ and 95 % Confidence Intervals [Lower Bound, Upper Bound]
Medial anterior outer tibial cartilage (aMT-O)	0.06 [−0.55, 0.62]
Medial central outer tibial cartilage (cMT-O)	−0.22 [−0.71, 0.42]
Medial posterior outer tibial cartilage (pMT-O)	−0.21 [−0.71, 0.43]
Medial anterior inner tibial cartilage (aMT-I)	−0.30 [−0.75, 0.35]
Medial central inner tibial cartilage (cMT-I)	−0.67 [−0.90, −0.14]*
Medial posterior inner tibial cartilage (pMT-I)	−0.15 [−0.68, 0.48]
Lateral anterior outer tibial cartilage (aLT-O)	−0.30 [−0.75, 0.35]
Lateral central outer tibial cartilage (cLT-O)	0.28 [−0.37, 0.74]
Lateral posterior outer tibial cartilage (pLT-O)	0.01 [−0.58, 0.59]
Lateral anterior inner tibial cartilage (aLT-I)	−0.42 [−0.81, 0.22]
Lateral central inner tibial cartilage (cLT-I)	−0.15 [−0.68, 0.48]
Lateral posterior inner tibial cartilage (pLT-I)	−0.04 [−0.61, 0.56]

* 95 % confidence intervals do not cross 0.

## Discussion

Our hypothesis was partially supported by our results, which indicated that slower habitual walking speed was moderately associated with greater cartilage strain in the central inner ROI of the medial tibia (Fig. 2). Conversely, all other associations between walking speed and tibial cartilage strain in any other tibial ROI were weak to moderate and had 95 % confidence intervals that crossed 0 (Table 3). Small-to-moderate association magnitudes (between 0.30 – 0.42, Table 3) between slower habitual walking speed and greater cartilage strain were exhibited in the anterior outer and anterior inner ROI of the lateral tibial cartilage and the anterior inner ROI of the medial tibial cartilage, suggesting that slower walking speeds may be related to cartilage strain over more tibial ROI at smaller magnitudes than our study was powered to detect. Overall, our study demonstrates a measurable *in-vivo* link between slower habitual walking speed and greater cartilage strain, which provides further rationale for evaluating the mechanistic link between modifying habitual walking speed to influence important biophysical changes in articular cartilage health.

Only the central inner ROI of the medial tibia demonstrated a moderate association with 95 % confidence intervals that did not cross zero between greater cartilage strain and slower walking speed (ρ = −0.67, Fig. 2). Previous work has demonstrated a higher propensity to develop KOA in the medial tibiofemoral compartment [30] and slower walking speeds are associated with early compositional changes in the medial tibiofemoral articular cartilage in the first 6 months post-ACLR [10]. Furthermore, tibial strain is similarly increased following walking in the medal tibia in patients with non-surgical ACL injuries compared to the uninjured limb [20]. While we must caution against the interpretation of a robust association between walking speed and cartilage strain from an association in a single ROI, our results indicate that future studies should be powered to detect small-to-moderate associations between these variables. We did find small-to-moderate association magnitudes in two ROI of the lateral tibial condyle (anterior outer and anterior inner ROI of the lateral tibial cartilage; Table 3) suggesting that studies with a greater number of participants may conclude that slower walking speed is linked to greater cartilage strain in both the medial and lateral compartments.

A previous study reported that uninjured individuals who walk with slower speeds demonstrate less cartilage strain in the medial, but not the lateral tibia [19] opposing the directionality of the relationship reported in the current study of individuals with ACLR (i.e., slower walking speed and greater medial tibial cartilage strain). The average medial tibial strain (2.69±4.64 %) in our participants with a history of ACLR was slightly smaller compared to uninjured individuals (3 ± 2 %) [19] from the prior study, but slightly larger in the lateral tibia (ACLR participants from current study: 3.34±4.34 %; uninjured participants from prior study: 3 ± 1 %) [19]. However, it is difficult to determine if these small differences are clinically meaningful and caution should be exercised when comparing these tibial strain values due to methodological differences between studies. Multiple biological and biomechanical changes are occurring simultaneously following ACL injury and ACLR [21,31,32] that may influence the association between habitual walking speed and articular cartilage strain and may account for the differing relationships reported in uninjured controls. Multiple studies have demonstrated that ACL injury is associated with greater pro-inflammatory cytokines and biochemical changes associated with cartilage breakdown [31] and deleterious changes in cartilage composition (i.e., decreased proteoglycan density and disorganized type-II collagen structure) [33]. Slower walking speed is also associated with deleterious biochemical changes [9] and worse cartilage composition following ACLR [10], suggesting that there may be a complex interaction between multiple biological and biophysical factors. We can speculate walking speed may slow as an indicator of deleterious biological breakdown of the joint tissue in response to injury; subsequently, the slowed walking speed may negatively affect the nature by which the cartilage is loaded – resulting in aberrant loading of already weaken cartilage tissue and hastened joint tissue breakdown. Future research should incorporate biological factors when determining the mechanistic link between slower walking speeds and greater cartilage strain.

In healthy individuals, walking speed remains relatively stable for the first 7 decades of the life [34]; thereby, it has been suggested that habitual walking speed should be collected as a vital sign of overall change in health status [35]. Slower habitual walking speed is a sign of the underlying development or progression of a myriad of poor health outcomes (e.g. type-II diabetes [36], cardiovascular disease [37], depression [38]). Slower walking speeds are predictive of future onset and progression of KOA in cohorts of individuals who are > 50 years of age [7,8,39] and deleterious changes in tibiofemoral articular cartilage composition and joint tissue biology in younger individuals (e.g., 18–35 years old) with an ACL injury [9,10]. For many conditions (e.g. type-II diabetes [36], cardiovascular disease [37], depression [38]), it is hypothesized that slower habitual walking speed is an indicator of underlying disease status, while the rationale for slower habitual walking speed contributing to the progression of the disease status is limited. Our observational study cannot determine causality that slower walking speeds are mechanistically linked to greater cartilage strain post-ACLR. However, it can be hypothesized that slower walking speed may have an effect on the development of KOA since articular cartilage is directly influenced by the nature of the joint loading applied in a time-dependent manner [12]. We can speculate that slower walking speeds reported in individuals post-ACLR [21] contribute to greater duration of cartilage compression during a single step, especially during the midstance phase of walking [21,40], leading to greater cartilage strain in the central inner ROI of the medial tibia. Caution should be taken with this interpretation though because individuals who walk at slower speeds over a standardized number of steps (i.e., 3000 steps) dictated by our standardized walking protocol will walk for longer durations. Therefore, it is unclear if this association may be driven by the total duration of the task (i.e. length of time a person walks) or the cumulative effect of multiple compression events (i.e., number of steps) where loading is applied in a specific manner.

While our study is novel and provides important information regarding the role of walking speed on articular cartilage strain following ACLR, there are limitations that should be considered to inform future research. Our study was powered to detect moderate-to-strong associations (ρ = 0.7) between walking speed and cartilage strain. There may be associations between walking speed and cartilage strain that are small-to-moderate in magnitude (Table 3) that we were not powered to detect but could have been deemed meaningful. It should be considered that relatively small associations may influence joint tissue health as the tissues may be loaded between 6000 and 12,000 times per day in two-thirds of the ACLR population [41]. We sampled a heterogenous group of patients from one large university orthopedic clinic at different time points post-ACLR, with different graft types and history of concomitant injury (e.g. meniscal injury, chondral injury, Table 1) and we were not powered to evaluate the effects of these variables on our primary analyses. The generalizability of our results should be interpreted with caution. Future studies with larger sample sizes from different regions and orthopedic clinics should evaluate the effects of confounding variables on the association between walking speed and cartilage strain to improve the generalizability of the results. Similar to the current study in individuals post-ACLR, a previous study completed in uninjured individuals focused on the relationship between walking speed and tibial articular cartilage strain, specifically [19]. Ultrasound studies have demonstrated that slower walking speeds in uninjured individuals are associated with greater articular cartilage strain across a small ROI of the femoral trochlear during a 30-minute acute walking protocol suggesting that the biomechanical response of the patellofemoral joint may also relate to walking speed [16]. Future work should seek to incorporate cartilage strain measurements from the femur and patella as well as in comparison to the participant’s uninjured limb to best determine the effect of walking speed on tissue of all articulating surfaces affected by ACL injury and ACLR. Finally, our study only evaluated the association between habitual walking speed and cartilage strain, and it remains unknown if the walking speed selected by the participants was significantly different from pre-injury speed. Future studies should examine the effect of modulating walking speed to determine the mechanistic effect of speed on cartilage strain.

We can conclude that slower habitual walking speed was moderately associated with greater cartilage strain in the central inner ROI of the medial tibia. Small-to-moderate association magnitudes (between 0.30 – 0.42) between slower habitual walking speeds and greater cartilage strain were exhibited in the anterior outer and anterior inner ROI of the lateral tibial cartilage and the anterior inner ROI of the medial tibial cartilage but had 95 % confidence intervals that crossed zero. The directionality of the association between habitual walking speed and cartilage strain may be specific to individuals post-ACLR due to the complex interaction between biomechanical and biological changes following injury and surgery, but future research is needed to confirm mechanistic links.

## Role of the funding source

The project described was supported by the Thurston Arthritis Research Center Core Center for Clinical Research Pilot and Feasibility Studies (P30AR072580).

## Declarations of interest

The project described was supported by the Thurston Arthritis Research Center Core Center for Clinical Research Pilot and Feasibility Studies (P30AR072580).

## Declaration of generative AI and AI-assisted technologies in the writing process

Generative AI and AI-assisted technologies were not used during the writing process of this manuscript.

## Availability of data and materials

The data will be available to those who submit a formal written request to the Principal Investigator (Dr. Brian Pietrosimone) and commit to only using the data for researcher purposes unplanned by the investigators.

## CRediT authorship contribution statement

**Caroline Lisee:** Conceptualization, Methodology, Formal analysis, Investigation, Data curation, Writing – review & editing, Funding acquisition. **David Lalush:** Conceptualization, Methodology, Formal analysis, Data curation, Writing – review & editing, Funding acquisition. **Cortney Armitano-Lago:** Methodology, Investigation, Writing – review & editing. **Elizabeth Bjornsen:** Methodology, Investigation, Writing – review & editing. **Jason R. Franz:** Conceptualization, Methodology, Writing – review & editing, Funding acquisition. **Jeffrey T. Spang:** Conceptualization, Investigation, Writing – review & editing, Funding acquisition. **Matthew E. Dausch:** Methodology, Formal analysis, Data curation, Writing – review & editing. **Brian Pietrosimone:** Conceptualization, Methodology, Writing – original draft, Supervision, Funding acquisition.

## Declaration of competing interest

None
